# Risk factors for development of new skin neoplasms in patients with past history of skin cancer: A survival analysis

**DOI:** 10.1038/s41598-018-33763-7

**Published:** 2018-10-24

**Authors:** Ana Filipa Duarte, Bernardo Sousa-Pinto, Eckart Haneke, Osvaldo Correia

**Affiliations:** 1 Centro de Dermatologia Epidermis, Instituto CUF, Porto, Portugal; 2 MEDCIDS - Department of Community Medicine, Information and Health Decision Sciences, Faculty of Medicine, University of Porto, Porto, Portugal; 3 CINTESIS - Center for Health Technology and Services Research, University of Porto, Porto, Portugal; 4 Basic and Clinical Immunology Unit, Department of Pathology, Faculty of Medicine, University of Porto, Porto, Portugal; 5 Department of Dermatology, Inselspital, University of Bern, Bern, Switzerland; 6 Dermatology Clinic Dermaticum, Freiburg, Germany; 7 Department of Dermatology, University of Ghent, Ghent, Belgium

## Abstract

We conducted a retrospective study aiming to assess the risk, and associated risk factors, of developing subsequent skin cancers after having a first diagnosis of skin cancer. We included all patients with biopsy-proven skin cancer attending a dermatology clinic between July 2007 and July 2017. We assessed the frequency of new skin cancers, as well as potential demographic and clinical factors significantly associated with occurrence of such neoplasms, that were identified by means of a survival analysis. We analyzed 969 patients with a total of 1584 skin neoplasms (1122 basal cell carcinomas (BCC), 310 squamous cell carcinomas (SCC), 143 melanomas and 9 other neoplasms). 165 patients (17.0%) developed subsequent skin neoplasms. Factors identified in multivariable models to be significantly associated with development of new skin cancers included older age (adjusted HR = 1.04 *per* year; 95%CI = 1.02–1.05; *p* < 0.001), and presence of synchronous neoplasms (adjusted HR = 2.25; 95%CI = 1.61–3.14; *p* < 0.001). Having a history of a BCC was significantly associated with development of new BCC (adjusted HR = 1.63; 95%CI = 1.05–2.54; *p* = 0.030), while having a previous SCC was associated with occurrence of subsequent SCC (adjusted HR = 3.60; 95%CI = 1.93–6.72; *p* < 0.001). These findings point to the importance of careful follow-up (e.g., skin self-examination and full body examination) of skin cancer patients.

## Introduction

The skin is the most frequent location of primary malignant neoplasms^1–3^. Moreover, skin cancer incidence is increasing worldwide^4–6^. While malignant melanoma (MM) has a higher lethality than other types of skin cancer, non-melanoma skin cancer (NMSC) - including basal cell carcinoma (BCC) and squamous cell carcinoma (SCC) – is far more common and is also associated with substantial morbidity, loss of function, disfigurement, and costs^7,8^.

The follow up of patients with personal history of skin cancer has been subject of debate, particularly concerning its frequency and duration^9^. In fact, it is known that when a patient is diagnosed for the first time with skin cancer, there is an increased risk of developing subsequent skin neoplasms^10–12^. However, this increased risk has been insufficiently quantified and associated epidemiological factors have not been properly identified.

Therefore, this study aims to evaluate the risk of developing a subsequent skin neoplasm after having a first diagnosis of skin cancer, as well as to identify potential factors associated with an increased risk of new neoplasms.

## Results

Within the studied period, we assessed 969 different patients with a total of 1584 skin neoplasms (including 1122 BCCs, 310 SCCs, 143 MMs and 9 other neoplasms); 675 patients had a single skin neoplasm, while 294 individuals had more than one skin cancer. The median “free-of-new-neoplasms” follow-up time was of 45 months. Overall, 165 patients (17.0%; 95% CI: 14.6–19.4%) presented metachronous tumours after having been diagnosed with the first one. Moreover, a total of 178 patients had synchronous neoplasms. In most cases, the type of these new neoplasms was the same histological type as the first assessed lesion. Therefore, of all assessed patients, a total of 133 developed new BCCs (13.7%; median follow-up time until a new BCC was diagnosed: 19 months), 48 developed new SCCs (5.0%; median follow-up time: 19 months), and 7 developed new MMs (0.7%; median follow-up time: 50 months). Overall, the median follow-up time was of 17 months for patients who developed new skin neoplasms *versus* 11 months for the remainder. Patients’ demographic and clinical characteristics at first assessment are described in Table 1. Overall, patients’ age ranged between 17 and 102 years. Patients who developed new neoplasms were in average older than the remainder (69 *versus* 63 years, *p* < 0.001) and had a higher expression of synchronous neoplasms at first diagnosis (33.3% *versus* 15.3%; *p* < 0.001).

**Table 1:** Characteristics of patients when they were first diagnosed with a skin neoplasm, and comparison between those who subsequently developed other skin neoplasms (“patients with new neoplasms”) versus those who did not (“patients with no new neoplasms”).

	All patients (*n* = 969)	Patients with no new neoplasms (*n* = 804)	Patients with new neoplasms (*n* = 165)	*P* value^a^
Age^b^ – mean (SD)	64.0 (16.1)	63.0 (16.6)	69.0 (12.7)	<0.001†
Gender – *n* (%)				
Male	458 (47.3)	375 (46.6)	83 (50.3)	0.391*
Female	511 (52.7)	429 (53.4)	82 (49.7)	
Histological type of neoplasms^b^ – *n* (%)				
Basal cell carcinoma	665 (68.6)	548 (68.2)	117 (70.9)	0.488*
Squamous cell carcinoma	202 (20.8)	157 (19.5)	45 (27.3)	0.026*
Melanoma	130 (13.4)	121 (15.0)	9 (5.5)	0.001*
Other	8 (0.8)	6 (0.7)	2 (1.2)	0.630**
Anatomical location of neoplasms^b^ – *n* (%)				
Head and neck	549 (56.7)	450 (56.0)	99 (60.0)	0.341*
Trunk	241 (24.9)	197 (24.5)	44 (26.7)	0.558*
Upper limb	161 (16.6)	126 (15.7)	35 (21.2)	0.082*
Lower limb	121 (12.5)	95 (11.8)	26 (15.8)	0.163*
Infiltrative neoplasms^b^	212 (21.9)	180 (22.4)	32 (19.4)	0.397*
Presence of synchronous neoplasms^b,c^	178 (18.4)	123 (15.3)	55 (33.3)	<0.001*

^†^Two-samples independent t-test; *Chi-square test; **Fisher’s exact test.

^a^*P* values obtained by comparing patients with no new neoplasms *versus* patients with new neoplasms; ^b^Variables assessed at the time of the first skin cancer diagnosis; ^c^Includes 114 patients (64.0%) with synchronous BCC only, 27 patients (15.2%) with synchronous SCC only, 26 patients (14.6%) with synchronous BCC and SCC, 7 patients (3.9%) with synchronous BCC and melanoma, and 4 patients (2.2%) with synchronous melanoma only.

We performed Cox proportional hazard regression analysis to estimate variables at first assessment potentially associated with development of new skin neoplasms (there were no significant differences in follow-up time regarding any of the independent variables tested). In univariable analyses, diagnosis of an SCC, older age and presence of synchronous neoplasms were significantly associated with development of new skin neoplasms. However, in multivariable models, significant associations were only observed for age (adjusted HR = 1.04 *per* year; 95%CI = 1.02–1.05; *p* < 0.001) and presence of synchronous neoplasms (adjusted HR = 2.25; 95%CI = 1.61–3.14; *p* < 0.001) (Table 2).

**Table 2:** Results of the univariable and multivariable Cox proportional hazard regression analyses of all new skin neoplasms survival, new basal cell carcinomas (BCC) survival, and new squamous cell carcinomas (SCC) survival.

	Univariable analysis: HR (95%CI); *p* value	Multivariable analysis: HR (95%CI); *p* value
**All skin neoplasms**		
Diagnosis of a BCC^a^	1.30 (0.93–1.83); 0.117	—^c^
Diagnosis of a SCC^a^	1.67 (1.19–2.36); 0.005	1.03 (0.71–1.48); 0.895
Age^a^	1.04 (1.03–1.05); <0.001	1.04 (1.02–1.05); <0.001
Male gender^b^	1.27 (0.93–1.73); 0.128	1.09 (0.80–1.49); 0.590
Infiltrative neoplasm^a^	0.78 (0.53–1.14); 0.189	—
Presence of synchronous neoplasms^a^	2.92 (2.11–4.06); <0.001	2.25 (1.61–3.14); <0.001
**BCC**		
Diagnosis of a BCC^a^	1.96 (1.30–2.97); <0.001	1.63 (1.05–2.54); 0.030
Age^a^	1.04 (1.03–1.05); <0.001	1.04(1.02–1.05); <0.001
Male gender^b^	1.36 (0.97–1.91); 0.079	1.18 (0.84–1.67); 0.349
Infiltrative neoplasm^a^	0.68 (0.43–1.06); 0.076	0.71 (0.44–1.15); 0.161
Presence of synchronous neoplasms^a^	2.98 (2.07–4.28); <0.001	2.27 (1.55–3.32); <0.001
**SCC**		
Diagnosis of a SCC^a^	5.77(3.26–10.21); <0.001	3.60 (1.93–6.72); <0.001
Age^a^	1.07 (1.04–1.09); <0.001	1.04 (1.01–1.07); 0.002
Male gender^b^	1.40 (0.80–2.48); 0.240	—
Infiltrative neoplasm^a^	1.55 (0.84–2.86); 0.172	—
Presence of synchronous neoplasms^a^	2.64 (1.46–4.78); 0.003	1.93 (1.05–3.55); 0.035

HR = Hazard ratio; 95% CI = 95% confidence interval.

^a^Variables assessed at the time of the first skin cancer diagnosis; ^b^Female gender was defined as reference category; ^c^Diagnosis of a BCC was not introduced in the multivariable model, as diagnosis of a SCC was already introduced.

**Figure 1:**
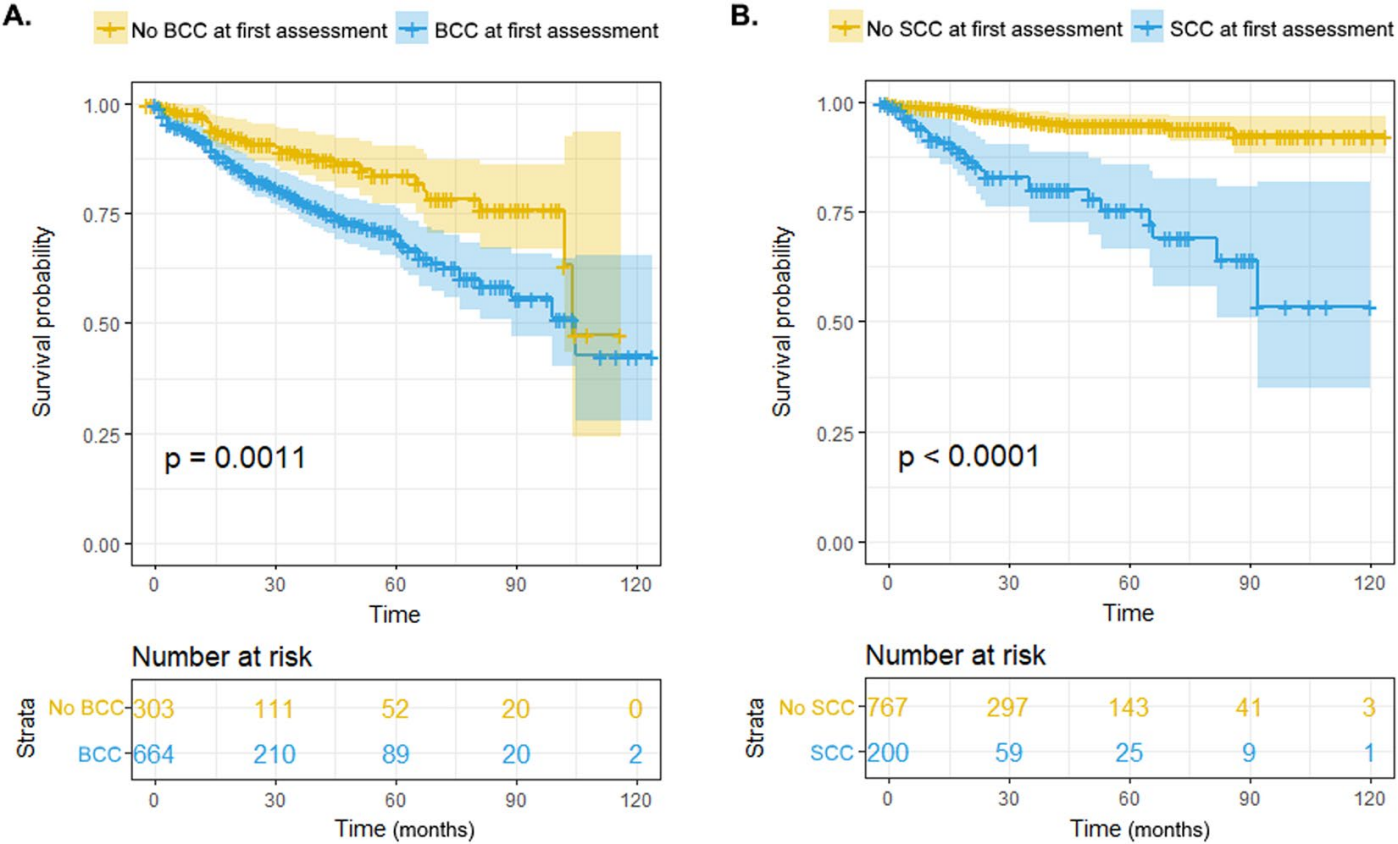
Kaplan-Meier survival curves for diagnosis of new basal cell carcinomas (BCC) comparing patients who presented with BCC at first diagnostic assessment *versus* those who did not (**A**), and for diagnosis of new squamous cell carcinomas (SCC) comparing patients which presented with SCC at first diagnostic assessment *versus* those who did not (**B**). *P* values were obtained using log-rank test.

Kaplan-Meier curves for development of new BCC and SCC are depicted in Fig. 1. Patients with BCC at first assessment had lower new BCC-free survival than the remainder (*p* = 0.001); accordingly, patients with SCC at first assessment had lower new SCC-free survival than the remaining patients (*p* < 0.001) (Fig. 1). In multivariable Cox regression models, predictive factors significantly associated with development of new BCC included presence of a BCC at first assessment (adjusted HR = 1.63; 95%CI = 1.05–2.54; *p* = 0.030), age (adjusted HR = 1.04 *per* year; 95%CI = 1.02–1.05; *p* < 0.001) and presence of synchronous neoplasms at first assessment (adjusted HR = 2.27; 95%CI = 1.55–3.32; *p* < 0.001) (Table 2). Regarding SCC, significant associations were also observed for age (adjusted HR = 1.04 *per* year; 95%CI = 1.01–1.07; *p* = 0.002) and presence of synchronous neoplasms at first assessment (adjusted HR = 1.93; 95%CI = 1.05–3.55; *p* = 0.035), as well as for presence of an SCC at first assessment (adjusted HR = 3.60; 95%CI = 1.93–6.72; *p* < 0.001) (Table 2).

Considering only patients diagnosed with a BCC within their first assessment, having a non-superficial BCC was associated with a decreased risk of developing a new skin cancer (HR = 0.66; 95%CI = 0.46–0.96; *p* = 0.027), including a new BCC (HR = 0.68; 95%CI = 0.46–1.00; *p* = 0.048). By contrast, in those patients, having a BCC located outside the head and neck at first assessment was significantly associated with development of new skin neoplasms (HR = 1.46; 95%CI = 1.01–2.11; *p* = 0.041), particularly new BCC (HR = 1.54; 95%CI = 1.05–2.28; *p* = 0.027). These findings are inter-related, as most superficial BCC (67.8%) were found to be located outside the head and neck, while most non-superficial BCC (73.7%) were located in the head or neck.

## Discussion

In this study, we assessed 969 patients with skin cancer, observing that 17.0% of them developed subsequent skin neoplasms. Advanced age and detection of synchronous neoplasms at first diagnosis were identified as risk factors for the development of new skin neoplasms. In addition, patients with a past history of BCC or SCC were found to be at an increased risk of developing skin neoplasms of the same histological type; this association, however, was stronger for SCC than for BCC.

Previous studies have reported the 3-year cumulative risk of a BCC or an SCC to be at least 10-fold increased when compared with the general population^9^. Our data showed a median of one year and a half for the development of a second NMSC. Meta-analysis data revealed that the 3-year cumulative risk of an SCC is 18% after a first SCC and 6% after a BCC; while the 3-year cumulative risk of BCC is 44% after a first BCC or an SCC^9^. In a more recent meta-analysis, the highest risk was found for the development of subsequent cutaneous neoplasms of the same type^10^, as reported with our data. Patients who develop BCC and SCC have lower incidence of new BCC when compared to patients who have BCC only, possibly due to sun exposure patterns favoring SCC or to innate immunity trigger^13^.

We observed few patients developing a MM as a second neoplasm, and therefore we were not able to explore the potential risk factors for the occurrence of a second MM. However, the median follow-up time to the diagnosis of metachronous MM was above 4 years. Previous studies have reported that among MM patients, the incidence of a second primary MM ranges from 0.2% to 8.6%^14^, being higher in younger people^15^.

This study has some limitations, particularly as it has a retrospective design and is based on a single private healthcare institution which might not be representative of the whole country. Nevertheless, the demographic and clinical characteristics of the patients assessed are mostly consistent with those described in the literature, particularly concerning their mean age, gender distribution and proportion of BCC/SCC and NMSC/MM^4,16,17^. Another limitation concerns the fact that the follow-up period varied according to the time of entry into the study, with participants who did not develop a new skin cancer having a lower median follow-up period than those who did, possibly resulting in an under-estimation of the incidence of metachronous neoplasms (some participants might have been identified as not developing new skin tumors simply because they were early losses to follow-up). Finally, this study did not include the analysis of SCC precursor lesions, namely actinic keratosis.

The assessment of all patients with confirmed diagnosis of skin cancer observed in a 10 year-period is an important strong point of this work. In addition, we performed univariable and multivariable Cox regression analyses, allowing adjusted results for each tested independent variable to be obtained.

Not only did we observe a high incidence of new skin neoplasms, but also a high frequency of patients presenting with more than one neoplasm at first diagnosis. In fact, almost one third of the patients that developed metachronous neoplasms had two or more skin neoplasms at first diagnosis. Skin cancer is an etiologically complex disease with ultraviolet radiation exposure, phenotype and genotype being probably involved in the risk of synchronous and metachronous lesions^18,19^. This fact highlights the importance of registering all skin cancers, even if having the same histological type and/or the same location. Unfortunately, NMSC has been widely disregarded in national cancer registries, possibly due to their extremely high frequency and low lethality^7,8^, impairing an appropriate understanding of skin cancer epidemiology and costs.

These results, along with the finding that the presence of synchronous neoplasms is associated with a higher risk of developing metachronous tumors, indicate the extreme importance of a full skin body examination^20^ and of a careful follow-up of skin cancer patients. Informing skin cancer patients about their risk of developing another skin cancer, together with teaching them skin self-examination every 1-2 months is mandatory, but it can cause anxiety^21^ if not accompanied by an easy access to physicians.

Nevertheless, the adequate follow-up frequency and duration for skin cancer patients remains debatable. An at least annual full skin examination for 3 to 5 years for NMSC, and every 3 to 6 months, depending on stage^22^, for 5 years for MM patients is desirable^23^. While follow-up surveillance of MM patients should be lifelong (although there is no international agreement for follow-up guidelines after 5 years from diagnosis)^15^, follow-up of NMSC for more than 3–5 years (3 years for BCC and 5 years for SCC) is currently not recomended^9,24^. This study’s results, however, might justify further studies to establish the best follow-up protocol for NMSC according to patients’ risk factors, while also emphasizing the importance of a comprehensive examination of these patients, assisted by dermoscopy^25^. This assessment is particularly important as early detection and treatment of MM and NMSC can reduce morbidity (and, particularly in the case of MM, mortality)^26,27^, bring a better health for the population^28^ and reduce the financial burden for the society^29–35^.

In conclusion, we observed that patients with history of skin cancer had a risk of 17.0% of developing new skin neoplasms, with advanced age and detection of synchronous neoplasms at first diagnosis identified as risk factors for the development of metachronous lesions. These findings point to the importance of total skin examination and careful follow-up of all skin cancer patients, including those with NMSC. Further studies are needed to evaluate the best follow-up protocols according to patients’ risk factors and type of neoplasms diagnosed. Meanwhile, teaching skin self-examination (including the most frequent benign lesions) and improving the general practitioners diagnosis accuracy might improve the referral to dermatology centers, which might decrease morbidity, mortality and costs.

## Methods

We performed a retrospective cohort study assessing all patients diagnosed with at least one biopsy-proven skin neoplasm between July 2007 and July 2017 in a single institution (a private dermatology center in the North of Portugal). For all skin neoplasms, we recorded their histological type (BCC, SCC, MM or other), their anatomical location (categorized as “head or neck” *versus* “outside the head and neck”) and whether they were or not infiltrative (for BCC)/invasive (for SCC and MM). Additionally, we recorded whether each patient presented with a single or with multiple skin neoplasms simultaneously (synchronous neoplasms) at their first assessment. For BCC, we also took into account its subtype – BCC subtypes were categorized as “superficial” (including superficial and multifocal neoplasms) or “non-superficial” (including nodular, infiltrative, and ulcerated neoplasms).

The skin cancer diagnosis was clinically performed by a senior dermatologist after full body examination, and was assisted by dermoscopy followed by a biopsy for histopathological diagnostic confirmation. Full body examination is performed in the majority of patients attending our dermatology center irrespectively of their initial complaint. Patients with genetic syndromes associated with multiple skin cancers and immunosuppressed by organ transplantation were not included. Follow-up periods were established according to the skin cancer type – every 6 six months for MM in the first 5 years, and thereafter annually; every 6 months for NMSC in the first 3 years, and thereafter annually.

We assessed whether the included patients developed new cutaneous neoplasms (metachronous tumors) until July 2017 and recorded the diagnosis date for the first new neoplasm of each histological type. We calculated the skin cancer survival free time, corresponding to the time period between the date of the first diagnosed skin neoplasm(s) and either (i) the date of the next skin neoplasm diagnosed after the first one(s) or (ii), if no new skin neoplasms were detected after the first one(s), the date of the last follow-up/clinic visit. Accordingly, we also calculated BCC and SCC specific survivals, respectively based on the dates of the first BCC and of the first SCC diagnosed after the first skin neoplasm(s).

### Statistical analysis

Categorical variables were described using absolute and relative frequencies, while continuous variables were described using means and standard-deviations (SD). Chi-square test and Fisher’s exact test were used to compare categorical variables, whereas two-independent samples t-test was used with continuous variables.

We performed a Cox proportional hazard regression analysis in order to identify variables (assessed at the diagnosis of the first skin cancer(s)) significantly associated with occurrence of new skin neoplasms. Tested variables included patients’ gender, age, diagnosis of a BCC, diagnosis of a SCC, neoplasm anatomical location, presentation of synchronous neoplasms, and presence of at least one infiltrative/invasive neoplasm. Firstly, we performed univariable analyses, and then we included all variables with at least marginal association (*p* < 0.150) with the dependent variable in a multivariable model. Similar analyses were specifically performed for the development of new BCC and new SCC. We performed a subanalysis considering only patients with initial diagnosis of BCC, in order to assess whether the BCC subtype was significantly associated with development of new neoplasms. Results were presented using hazard ratios (HR) and 95% confidence intervals (CI).

Concerning the occurrence of new BCC, we also obtained Kaplan-Meier survival curves comparing patients who presented with a BCC at first diagnosis *versus* those who did not. For the occurrence of new SCC, Kaplan-Meier survival curves compared patients who presented with a SCC at first diagnosis *versus* those who did not. Comparisons were assessed using the log-rank test.

*P* values lower than 0.05 were considered to be statistically significant. All statistical analyses were performed using software R (version 3.4.3); Cox regression was performed using package ‘survival’, and Kaplan-Meier curves were obtained using package ‘survminer’.

### Ethical approval and informed consent

All methods were carried out in accordance with relevant guidelines and regulations. Data analysed were fully anonymised and as such the Ethics Committee of Instituto CUF waived the need for approval.
